# Transmission of Aerosolized Seasonal H1N1 Influenza A to Ferrets

**DOI:** 10.1371/journal.pone.0024448

**Published:** 2011-09-19

**Authors:** Heather MacInnes, Yue Zhou, Kristine Gouveia, Jenna Cromwell, Kristin Lowery, R. Colby Layton, Michael Zubelewicz, Rangarajan Sampath, Steven Hofstadler, Yushi Liu, Yung-Sung Cheng, Frederick Koster

**Affiliations:** 1 Program in Infectious Disease, Lovelace Respiratory Research Institute, Albuquerque, New Mexico, United States of America; 2 Program in Aerosol Science, Lovelace Respiratory Research Institute, Albuquerque, New Mexico, United States of America; 3 Ibis Biosciences, A Subsidiary of Abbott Molecular Inc., Carlsbad, California, United States of America; 4 Program in Lung Cancer, Lovelace Respiratory Research Institute, Albuquerque, New Mexico, United States of America; 5 Program in Applied Science, Lovelace Respiratory Research Institute, Albuquerque, New Mexico, United States of America; Erasmus Medical Center, Netherlands

## Abstract

Influenza virus is a major cause of morbidity and mortality worldwide, yet little quantitative understanding of transmission is available to guide evidence-based public health practice. Recent studies of influenza non-contact transmission between ferrets and guinea pigs have provided insights into the relative transmission efficiencies of pandemic and seasonal strains, but the infecting dose and subsequent contagion has not been quantified for most strains. In order to measure the aerosol infectious dose for 50% (aID_50_) of seronegative ferrets, seasonal influenza virus was nebulized into an exposure chamber with controlled airflow limiting inhalation to airborne particles less than 5 µm diameter. Airborne virus was collected by liquid impinger and Teflon filters during nebulization of varying doses of aerosolized virus. Since culturable virus was accurately captured on filters only up to 20 minutes, airborne viral RNA collected during 1-hour exposures was quantified by two assays, a high-throughput RT-PCR/mass spectrometry assay detecting 6 genome segments (Ibis T5000™ Biosensor system) and a standard real time RT-qPCR assay. Using the more sensitive T5000 assay, the aID_50_ for A/New Caledonia/20/99 (H1N1) was approximately 4 infectious virus particles under the exposure conditions used. Although seroconversion and sustained levels of viral RNA in upper airway secretions suggested established mucosal infection, viral cultures were almost always negative. Thus after inhalation, this seasonal H1N1 virus may replicate less efficiently than H3N2 virus after mucosal deposition and exhibit less contagion after aerosol exposure.

## Introduction

Influenza is a highly contagious respiratory infection annually causing over 1.6 million infections [1] and 36,000 deaths [2] in the United States and causing periodic pandemics with increased mortality worldwide [3]. In spite of its major morbidity and mortality our knowledge of influenza transmission is poor [4], with little quantitative data available on exhaled virus from contagious hosts [5] or level of inhaled virus needed to initiate infection. Only one study in 1966 has measured the amount of inhaled seasonal influenza causing symptomatic infection in the susceptible human host [6]. Recent publications have quantified the aerosol virus dose of H3N2 and H5N1 strains causing infection in the ferret model [7], [8].

Quantitative information may help resolve the ongoing debate on the relative importance of the different modes of infection. Influenza transmission occurs by three modes that are difficult to separate in epidemiological analysis. Contact with a contagious individual or surfaces contaminated by virus and inhalation of airborne droplets from a cough or sneeze (droplet transmission) are thought to be common modes of transmission [9], [10]. Droplets greater than 5 µm in diameter briefly remain airborne and are arrested in the upper airways after inhalation, while particles less than 5 µm in diameter (droplet nuclei) are exhaled during normal breathing and talking as well as coughing and sneezing [11], are airborne for minutes to hours depending on size and density, and can reach deeper lung tissues [12] (aerosol transmission). Epidemiological evidence for aerosol transmission is accumulating [13], [14] but further support will derive from animal models.

Influenza models in the guinea pig and ferret approximate both the susceptibility and transmissibility of influenza. Transmission between guinea pigs has been used effectively to study relative transmission efficiency between strains and dependence on humidity and temperature [15], [16], but the guinea pig does not develop inflammatory disease with symptoms [17]. The ferret approximates human infection with respect to susceptibility, pathogenesis and transmission [7], [18]. In the ferret transmission of seasonal and pandemic 2009 H1N1 flu strains (but not HPAI H5N1 strains) can occur via non-contact modes [19], [20], [21]. A recent seminal study of seasonal H3N2 infection in the ferret has quantified exhaled virus and measured the minimal aerosol infectious dose [7].

We calculated in the present study the aerosol infectious dose for 50% of ferrets (aID_50_) for a seasonal human H1N1 influenza virus. We designed a two-chamber apparatus to nebulize virus into a controlled airflow environment permitting the measurement of airborne virus and directly estimate the dose of virus inhaled by the susceptible ferret. To approximate the typical duration of human exposure in classroom or workplace, we limited exposures of ferrets to 1 hour. To optimize the detection of very low levels of airborne virus, particles were collected on filters and in liquid impingers and analyzed by culture and two RT-PCR-based assays. Although evidence was found for viral replication in upper airway mucosa, viral cultures were negative suggesting that aerosol transmission resulting in contagious infection is not a characteristic of this particular H1N1 seasonal virus.

## Materials and Methods

### Influenza virus

A/New Caledonia/20/99 (H1N1) (NC99), a seasonal influenza strain included in the seasonal influenza vaccine for 7 years, was obtained from the Centers for Disease Control. The stock virus was passaged twice in 10-day old embryonated chicken eggs, once in Madin-Darby Canine Kidney (MDCK) cells and a final passage in eggs. Stock viruses had titers of 9.0×10^5^ focus-forming units (FFU) per mL on MDCK cells. Stocks were frozen at −80C until use, and were re-titered after each experiment to confirm the actual titer inoculated into the nebulizer.

### Exposure Apparatus

**Figure 1:**
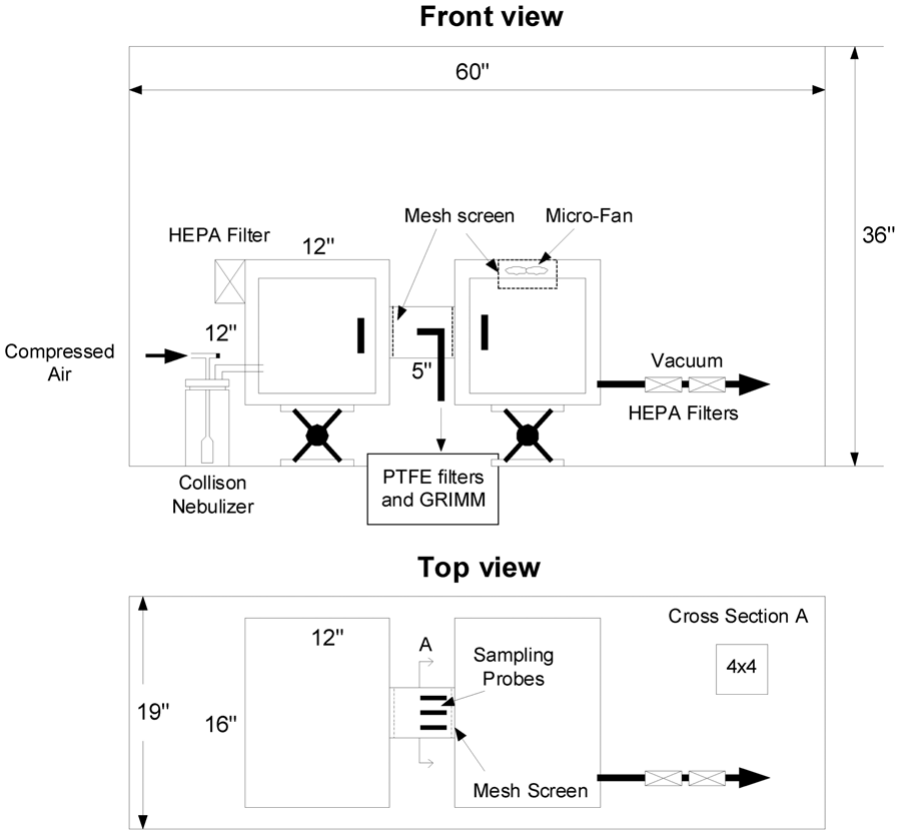
Schematic of the exposure chamber. The exposure apparatus is a pair of boxes with controlled airflow drawn through the HEPA filters in the origin (left) chamber wall to the vacuum line filters outside of the recipient chamber (right). The apparatus is housed in a Biosafety Cabinet class 2B for safety during ferret insertion and removal.

A small two-chamber Lexan exposure apparatus permitting quantitative monitoring of virus in aerosols artificially generated by nebulizer or exhaled by infected animals was designed and fabricated at Lovelace Respiratory Research Institute (LRRI) (**Figure 1**). The apparatus consisted of an ‘origin’ chamber 30 cm in length for each of the three dimensions, connected directly to a 6-jet Collison small particle generator (AGI, Inc.). The ‘origin’ chamber was connected through a 10×10 cm square metal tube 17.5 cm in length to the 30 cm^3^ “recipient” chamber with a small fan on the roof of the chamber to increase air circulation. The air was drawn by line vacuum through HEPA filters in the wall of the origin chamber at a rate of 12 L/min and exited the recipient chamber through HEPA filters. Nebulization into the origin chamber and through the metal tunnel permitted only particles less than 5 µm diameter to reach the recipient chamber (**Figure 1b**). Wire mesh screens at both ends of the tunnel prevented the ferret from contacting surface-contaminating virus on the walls of the tunnel and donor chamber. The entire apparatus was de-contaminated with bleach and alcohol after every exposure.

### Ferret Infection

All procedures were conducted under protocols approved by the Institutional Animal Care and Use Committee (IACUC) at LRRI, all facilities were accredited by the Association for Assessment and Accreditation of Laboratory Animal Care International (AAALAC), and guidelines for ferret housing, environment and comfort described in the Guide For The Care and Use of Laboratory Animals, Seventh Edition, National Research Council, were strictly adhered to. Ferrets weighing 800 to 1200 g were purchased from Triple F Farms (Sayre, PA) and housed in BSL2 conditions for observation for at least 10 days and for serological confirmation by the hemagglutination inhibition assay for the absence of serum antibody to circulating influenza A and B strains. Ferrets were exposed to aerosolized virus for 1 h with air-flow rate of 5.7–6.0 L/min in the recipient chamber of the exposure apparatus. The ferrets were unanesthetized and spent variable amounts of time exploring the chamber and sleeping on the wire floor of the chamber. After exposure each ferret was housed in a separate cage in a ventilated rack (Allentown Cage Inc, Allentown, NJ) to prevent any direct or airborne contact with other ferrets. The upper respiratory tract was sampled by daily nasal wash and throat swab for 5 consecutive days beginning 24 hours after exposure. Nasal wash solution (1 mL of 0.1% bovine serum albumin fraction 5, 1% Antibiotic/Antimycotic (100 units penicillin G, 100 µg streptomycin sulfate, 0.25 µg amphotericin B, Invitrogen), in PBS) was flushed through each nare, retrieved in a collection cup and divided equally into RNABee for later RNA extraction and rapidly frozen for later viral culture. The throat was swabbed vigorously with a foam-tipped applicator moistened with nasal wash solution, vortexed for 20 sec in 1 mL nasal wash solution, divided as above, and frozen at −80°C until analysis. The criterion for ferret ‘infection’, i.e., evidence for continuing viral replication in upper airway mucosa, was all of the five daily samples positive by culture or RT-qPCR.

### Aerosol analysis

Viral aerosols were collected from the metal tube by a glass impinger (AGI, Inc) at a flow rate of 2–6 L/min. Either 1 sterilized gelatin filter (25 mm, SKC Inc., Eighty Four, PA), or 1–3 25 mm Polytetrafluoroethylene (PTFE) Teflon filters (2.0 µm pore size, SKC Inc.) were used each drawing an air flow rate of 2 L/min. Gelatin filters dried and shrunk during 1 h of nebulization, thus all filter data presented are from PTFE filters. Particle counting and sizing was performed with a 31-channel real time aerosol spectrometer (Model 1.109, GRIMM Technologies, Inc, Germany). The flow rate of the GRIMM optical counter was 1.2 L/min. In each exposure the Collison nebulizer and AGI contained 20 mL media consisting of MEM, 0.2% NaHCO_3_, 20 mM HEPES, 1% Antibiotic/Antimycotic, 20 mM L-Glutamine, 0.001% BSA, and 100 µL of Antifoam A (Sigma). Collison and impinger fluid samples were collected before and after nebulization and aliquoted into RNABee (1∶1 dilution) or frozen at −80°C and stored for viral culture. PTFE filters collected aerosolized virus at 2 L/min air flow for 1 h. After exposure the filters were vortexed for 20 seconds in 1 mL impinger fluid, the filter removed, and sample aliquoted equally into RNABee or frozen at −80°C for viral culture.

### Viral culture

Virus was titered by a microplaque focus-forming unit (FFU) assay. After the sample containing virus was incubated on MDCK monolayers with 1.25 µg/mL TPCK-trypsin at 35°C for 1 h, 1.2% Avicel was overlaid and incubated for 18 h at 35°C. After overlay removal and fixation with 4% paraformaldehyde for 30 min at 4°C, the wells were washed twice with 0.05% Tween-20 in PBS, incubated 20 min with 0.5% Triton-X-100 and 20 mM glycine in PBS, and washed twice. Plaques containing influenza antigen were identified by incubation with anti-influenza M1 antibody (MAB8251, Millipore) diluted 1∶1500 in 10% normal horse serum and 0.05% Tween-20 in PBS followed by secondary antibody diluted 1∶150 in 10% horse serum, stained with 0.4 mg/mL AEC in 0.05 M sodium acetate and 0.03% H_2_O_2_, and counted under 20× magnification.

### RNA Analysis

RNA in throat swab and nasal wash samples was extracted by vortexing 10 sec in 200 µL of BCP (1-Bromo-3-chloropropane), centrifugation at 13,000 rpm and 4°C for 10 min removal of the aqueous layer and extracted in the Kingfisher processor (Thermo Scientific) by the “Magmax Clear” protocol according to the manufacture's instructions. Large volume samples from nebulizer and liquid impingers were extracted using the Qiagen RNeasy Maxi kit adapted to contain 10 µL/mL of carrier RNA in the initial lysis buffer followed the manufacture's “RNA Clean-up” protocol and concentration by ethanol precipitation into a 60 µL volume. PTFE filter samples were extracted by Qiagen QIAamp Viral RNA kit with carrier RNA.

For analysis of RNA by the Ibis T5000™ Biosensor system, pan-influenza virus PCR primer sets were developed that are capable of amplifying all three influenza virus species (A, B, and C) and subtypes (HxNy) from different animal hosts (human, avian, swine, etc.) [22], [23], [24]. A panel of eight primers was selected comprising one pan-influenza primer pair, five influenza A-specific primer pairs, and two influenza B-specific primer pairs, thus targeting 6 of the 8 genomic RNA segments. Following initial RT-PCR, mass spectroscopy (ESI-MS) is used to “weigh” amplicons with enough accuracy to yield an unambiguous base composition (A_w_, G_x_, C_y_, T_z_) used to identify and/or differentiate a pathogen by interrogation of a large database of known influenza sequences. The LOD of the assay is 10 genome equivalents (GEq).

 For analysis of RNA by the standard RT-qPCR assay targeting one genomic RNA segment, samples were amplified in triplicate using either the ABI TaqMan One-Step RT-qPCR Master Mix Reagents Kit (33.3 nM primer and 13.3 nM probe) or the Qiagen Quantitect Virus Kit (333 nM primer and 80 nM probe). A standard curve for each plate contained nine log_10_ dilutions of influenza M1 gene RNA prepared by Invitrogen plasmid blunt-TOPO and Ambion Mega-short-Script kit. The M1 gene forward primer: TTC ACA GCA TCG GTC TCA CAG ACA, reverse: TCC AGC CAT CTG TTC CAT AGC CTT and probe: /56-FAM/AAC AGA ATG GTG CTG GCT AGC ACT /3BHQ_2/ (Integrated DNA Technologies, Coralville, IA). RT for 20′ at 50°C was followed by PCR at 95°C for 5 min, 40 cycles of 95°C for 15 sec, 60°C for 45 sec on an ABI 7900HT Fast Real-Time PCR System with ABI SDS 2.3 software. After manually setting the threshold for the midpoint for each standard curve, the mean slope was −3.5 for a mean of 93.07% efficiency, the mean y-intercept was 41.5, and the mean r^2^ was 0.998 for all plates. A sample was positive at a Ct of 35 when two out of three filters were positive yielding an LOD of 30 Genome Equivalents (Geq; genome copies)/sample.

### Calculation of Inhaled Virus

The viral RNA collected from the aerosol by the T5000 PCR-based assay was used to calculate virus inhaled according to the formula:
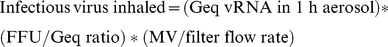
The FFU/Geq ratio was derived from filter collections of high levels of aerosolized virus with positive culture and viral RNA data (see **Table 1**). The fraction of available virus inhaled was determined by the minute ventilation divided by the airflow/min through the filter (2 L/min). Minute ventilation (MV) was estimated from unpublished data and literature reports, adjusted for ferret weight and sedation status. For anesthetized ferrets weighing 280 g and 560 g, tidal volumes (TV) have been reported as 4.0 and 6.0 mL, respectively [25], [26]. TV for our ferrets weighing between 0.8 and 1.2 kg was calculated as 9.0 mL according to the ¾-power relationship between body weight and lung volume. The breathing rate (RR) was observed to be a mean of 35/min (range 28–43), and the lack of sedation was adjusted by multiplying the TV by 1.5 (J Mauderly, personal communication), yielding a calculated MV (TV*RR*1.5) of 472 mL/min. The ID_50_ was estimated using Proc Probit in SAS 9.1 (Cary, NC).

**Table 1:** Comparison of impinger and PTFE filter efficiencies for culturable virus and viral RNA.

	Sample Source: impinger	Filter	Filter	Ratio^d^
Aerosol time^a^	FFU/collection^b^	FFU/collection^c^	GEq RNA/collection	FFU∶RNA
10 min	4.9 (0–19.6) E+03	4.9 (0.2–12.5) E+03	2.0 (0.9–3.9) E+06	1∶411
20 min	16.2 (0–28.5) E+03	12.2 (5.0–25.0) E+03	4.2 (2.3–7.6) E+06	1∶344
30 min	35.7 (19.5–49.3) E+03	4.0 (3.0–5.5) E+03	6.8 (4.1–9.1) E+06	1∶1705

a Virus NC99 was nebulized in 6 experiments with impinger alone and 9 experiments with impinger and PTFE filter collections in parallel. The mean (range) total dose nebulized was 1.8 (1.1–2.2)×10^7^ FFU, calculated by virus concentration in Collison at beginning of aerosol generation times fluid volume nebulized for each run.

b Impinger collection measured for 5.4 (range 5.3–5.5) L/min, reported as total FFU collected during interval of nebulization. N = 5 at each time point as one outlier value was removed from each group.

c PTFE filter collection combines two filters in parallel for total flow of 4.0 L/min, reported as total FFU and total genome equivalents (GEq) of RNA measured by T5000 assay.

d Ratio of group means of FFU and GEq RNA collected respectively.

## Results

### Aerosol Characteristics

**Figure 2:**
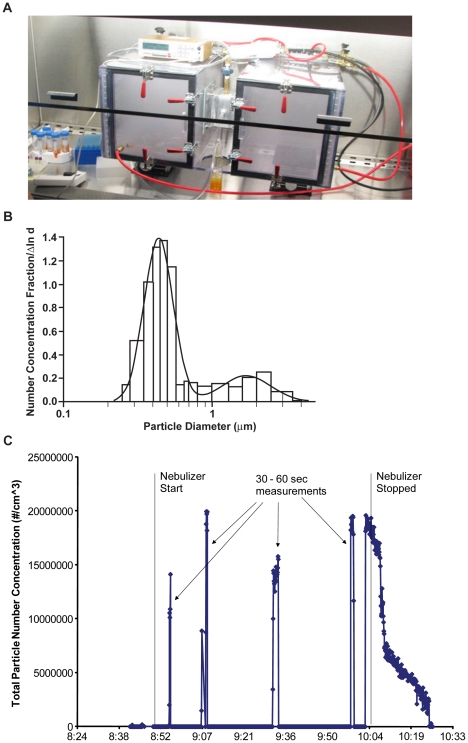
Characteristics of the aerosol exposure system. **a.** Photograph of exposure apparatus during nebulization with Collison generator inside the origin chamber shows the visible cloud of airborne particles in both the left (origin) and right (recipient) chambers. Placement of the Collison generator outside of the origin chamber, connected with 20 cm tubing, resulted in no visible suspended airborne particles in the chambers, and all subsequent experiments reported here had this configuration. The Grimm particle spectrometer is placed on top of the left chamber and the sampling port is located on top of the tunnel. **b.** The number particle size distribution with GRIMM optical counter. The fitted bimodal distribution has the median diameters of 0.44 and 1.70 µm, and geometric standard deviation of 1.25 and 1.46, for the two size modes, respectively. **c.** Particle number concentrations detected by Grimm laser-based particle counter sampled during four 30–60 sec intervals of the continuous one-hour nebulization.

In initial experiments the viral aerosol generated by the Collison nebulizer located outside the left chamber resulted in a visible cloud of particles in both chambers (**Figure 2a**). Relative humidity (RH) at the onset of nebulization was 40–45% and increased to 75–80% at the end of the hour. A diffusion dryer (In-Tox Products, Moriarty, NM) was then placed between the Collison and the chamber, resulting in no increase in RH, start RH mean (range) of 43% (41–48) compared to end RH mean 41% (31–53). The particle size showed a bi-modal distribution with a majority of particles in the sub-micrometer size (0.25 to 1 µm, median diameter 0.44 µm) and less than 5% between 1 and 5 µm (median diameter 1.70 µm) (**Figure 2b**). Particle concentration during aerosolization ranged from 1.4–2.0×10^7^ particles/mL (**Figure 2c**). The mean rate of fluid aerosolized by the Collison generator was 170 µl/min but the volume of each run varied from 5–14 mL at 60 min, so the amount of virus aerosolized was calculated according to the volume nebulized in each experiment. Thus exposures in our apparatus were exclusively due to virus airborne in particle nuclei applicable to aerosol transmission.

### Detection of Aerosolized Virus

**Figure 3:**
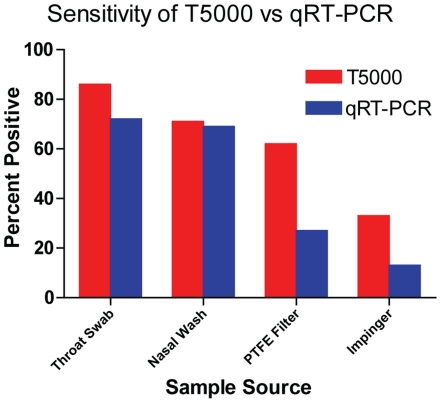
Comparison of sensitivity of the T5000 and RT-PCR Assays. Comparison of sensitivity of the assays for samples expected to contain relatively higher concentrations (3.0 to 7.0 log_10_ Geq) of viral RNA (throat swab and nasal wash) and relatively lower concentrations (1.0 to 4.0 log_10_ Geq) of viral RNA. The Ibis T5000 Influenza assay was not significantly more sensitive for detecting viral RNA in the airway specimens under the conditions of this experimental infection, but was significantly more sensitive in detection viral RNA in filter and impinger aerosol collection devices (Chi-Squared test, p<0.01).

We compared the relative sensitivity of two PCR-based methods, the Ibis T5000 assay that detects 6 genomic RNA segments for Influenza A, and a standard RT-qPCR assay that detects a single segment (M gene) in samples expected to contain high levels of virus (throat swab and nasal wash) and low levels of virus (airborne virus captured in liquid impinger and on PTFE filters (**Figure 3**). For all (N = 419) samples analyzed by both assays, 212 (50.6%) were positive by either one or both assays, and 85 samples (20.3%) were positive only by the T5000 assay. The single target RT-qPCR assay was never positive if the T5000 assay was negative, so the T5000 system was significantly more sensitive than the RT-qPCR assay (P = <0.0001, Fisher Exact Test). Among sample sources expected with high viral RNA content, there was no difference in sensitivity of the two assays, whereas for samples with low RNA levels (PTFE Filters and liquid impingers) the T5000 was significantly more sensitive (P = <0.001, Fisher Exact Test). There was a linear correlation between the calculated virus aerosolized and the viral genomes collected on the PTFE filters measured by the T5000 assay after one hour of collection (Linear Regression Test, Slope = 0.74, r^2^ = 0.22, F value = 9.93; data not shown). Examining only those experiments in which the recipient ferrets were infected, the linear relationship was more robust both for virus measured by T5000 assay (Linear Regression Test, slope = 0.775, r^2^ =  0.861, F = 37.3) and the RT-qPCR assay (slope = 1.57, r^2^ = 0.833, F = 20.0). Since the T5000 assay was more sensitive at low concentrations than the RT-qPCR assay, subsequent analysis used the T5000 data.

### Comparison of impinger and PTFE filter efficiencies

Different efficiencies of collection have been reported for impingers and filters, suggesting that each experimental set-up should be calibrated for relative efficiency. In our two-chamber exposure apparatus filters and impingers were compared during aerosolization of high levels of virus, with the RH controlled at approximately 40–45% with the diffusion dryer. Collections at 10, 20, and 30 min of viable virus were highly variable in the impinger (**Table 1**) and the collected virus did not appear to be additive with increasing time of collection. Collections of viable virus on the filters increased proportionately from 10 to 20 min, decreased at 30 min (**Table 1**) and were negative at 60 min (data not shown). Loss of culturable virus by 60 min may be due to inactivation by dessication, as the viral RNA on the filters increased proportionately with time. Viral RNA in the impinger liquid was consistently two log_10_ below that collected on parallel filters at each time interval (data not shown), possibly reflecting damage to RNA during bubble cavitation in the impinger. Although all methods were subject to considerable variance, the most consistent measure of aerosolized virus in the viral aerosol was viral RNA collected on PTFE filters.

Since initial ferret exposures to aerosolized NC99 virus resulted in very mild infection (see below **Table 2**), and since filters were uniformly negative for culturable virus after 60 min collection, we established an indirect approach to measuring inhaled virus by calculating a ratio between viable virus and viral RNA. The ratio of virus∶viral RNA ratio of 1∶344 (**Table 1**) after 20 min of collection on filters was used in subsequent analyses. In other sources of virus-containing samples highly variable ratios are calculated. The ratios in impinger collections varied from 1∶7 to 1∶0.16, reflecting both the variable virus collections as well as viral RNA levels two orders of magnitude lower than comparable filters. Ratios in the virus stock solutions in this study were 1∶44, and after 1 h of nebulization ratios of liquid remaining in the Collison were 1∶1050, probably due to loss of viability during bubble cavitation. Thus losses of viable virus in the Collison jets, on the walls of the first chamber and tunnel (confirmed by post-nebulization sampling), and loss of viability due to higher humidity resulted in less than 1% of the nebulized virus reaching the ferret chamber.

**Table 2:** Evidence of Infection among Ferrets exposed to high levels of nebulized NC99.

Exp.-Ferret	Log_10_ virus nebulized	Change In RH^a^	Aerosol vRNA GEq	Calculated^b^ Inhaled virus FFU/hour	Nasal wash^c^FFU RNA		HAI titer Day 12 pe
1 – A	7.00	45–80%	nd	-	0	nd	<20
-B	7.31	45–80%	nd	-	200	nd	320
-C	7.05	45–80%	nd	-	0	nd	320
-D	7.05	45–80%	nd	-	0	nd	<20
2 – A	7.08	42–75%	6.06	382	0	8.19	<20
-B	7.08	42–75%	6.27	2688	0	6.77	320
-C	5.06	41–53%	5.01	145	0	8.08	320
-D	7.11	41–53%	5.67	407	0	7.28	320

a For exposures to ferrets 2-C and 2-D, the diffusion dryer was inserted into the line exiting the Collison generator, reducing the humidity at the end of the hour nebulization.

b Calculation of inhaled virus based on measured viral RNA in aerosol inhaled during 60 min of aerosol exposure.

c Nasal wash was collected on day 2 post exposure (pe) in Exp 1, and daily in Exp 2; culture results are reported for the entire collection as FFU/mL; viral RNA is the peak titer on day 3 pe and is reported as GEq/collection.

### Detection of ferret infection after exposure

Ferrets were exposed to varying levels of nebulized virus to identify the aerosol ID_50_. No ferret exposed to nebulized virus expressed any symptoms of infection. In the first six ferrets exposed to high levels of aerosolized virus, humidity was not controlled during nebulization and evidence for infection was weak, with 5/8 (62%) seroconverting but only one ferret had culturable virus in the nasal wash (**Table 2**). For the last two ferrets in group 2 the humidity was reduced by insertion of the diffusion dryer downstream from the Collison. Exposure did not result in positive upper airway cultures, but levels of viral RNA were high (>10^4^ GEq/collection) for all nasal wash and throat swab samples for days 1 through 5 post exposure. This observation suggested that viral replication was established and continuing in upper respiratory tract mucosa but viable virus was not detectable in the nasal wash. Subsequent exposures to lower levels of aerosolized virus utilized the arbitrary definition of “infection” to be the presence of high levels of viral RNA (>7 log_10_ GEq/collection) for all 5 days post-exposure (**Table 3**). Lack of upper airway culturable virus could be due to a greater portion of virus inhaled into the lung resulting in seroconversion, but ferrets humanely euthanized 3 days post exposure had negative cultures and RNA assays in lung tissue. To support the notion that viral RNA present in washings for 5 days after exposure represented viral replication in the mucosa, even though cultures of nasal washes were negative, aliquots of 2×10^7^ FFU NC99 were exposed to ultraviolet light for 10 min rendering the preparation culture-negative on MDCK cells yet the RNA titer remained at approximately 10^9^ GEq/inoculation sample. Three ferrets were inoculated with 10^9^ GEq of viral RNA and nasal washes collected 1, 2, and 3 days later. Post-inoculation nasal washes were negative for detectable viral RNA, while the RNA inoculation aliquot stored at room temperature for 48 h in nasal wash solution remained detectable, suggesting that viral RNA is cleared from the respiratory mucosa within 24 h.

**Table 3:** Evidence of viral replication in ferrets exposed to low doses of aerosolized NC99.

Exp.- ferret	Target (a) Dose Nebulized	Aerosol virus On filter/h GEq/collection	Calculated (b) inhaled virus FFU/hour	Peak nasal wash virus RNA GEq/collection	Upper airway Viral replication
3 – A	1×10^3^	<2.0	<2	4.89	−
-B	1×10^3^	<2.0	<2	3.54	−
-C	1×10^5^	3.38	10	9.00	+
-D	1×10^5^	3.38	10	7.05	+
4 – A	1×10^4^	2.87	3	7.53	+
- B	1×10^4^	2.58	2	7.02	+
- C	1×10^4^	3.04	5	7.61	+
- D	1×10^4^	3.13	6	<2.0	−
- E	1×10^4^	2.86	3	<2.0	−
5 –A→E	1×10^3^ (N = 5)	<2.0	<2	<2.0	−

a. Actual dose calculated was within 30% of target dose.

b. Calculations based on viral RNA collected on PTFE filter during 60 min of aerosol exposure.

### Calculation of minimal ‘infectious’ dose (aerosol ID_50_)

All ferrets inhaling more than 10 virus particles had sustained viral RNA in upper airways but no positive cultures while 5/12 (42%) ferrets exposed to very low levels of viral aerosol had similar levels of sustained viral RNA (**Table 3**). The aID_50_ was estimated using Proc Probit in SAS to be 4 FFU with a 95% confidence interval between 1.8 and 186. Thus higher aerosol exposures to this seasonal H1N1 virus initiated seroconversion and mucosal replication, and lower exposures initiated at least mucosal replication but did not likely result in contagious infection.

## Discussion

Few studies have quantified the aID_50_ by nebulized influenza virus either in humans or in animal models. In a study performed over 40 years ago human volunteers were exposed to nebulized seasonal H3N2 influenza virus and the mean infectious aerosol dose was calculated to be 0.3 to 6 TCID_50_ [6]. The minimum infectious dose by intranasal drops was determined in susceptible volunteers to be substantially higher than the calculated aerosol ID_50_ [27]–[30]. Ferrets exposed to aerosolized H3N2 virus were readily infected by inhaled doses of approximately 1 TCID_50_ [7]. This low dose is comparable to the minimal infecting dose of 5 virus particles for an H3N2 strain in guinea pigs [15]. We report here that for an aerosolized seasonal H1N1 virus strain 4 FFU may initiate viral replication in the nasal mucosa, and exposure to more than 100 FFU resulted in seroconverson, neither higher nor low doses led to contagious infection. These results must be interpreted with respect to experimental design, limitations in aerosolized virus detection, and the characteristics of the influenza strain studied.

Exposure apparatus designs will likely affect measured transmission efficiencies. Our design restricted inhalation to droplet nuclei particles less than 5 µm in aerodynamic diameter by passage through a settling chamber and a tunnel. This design could have prevented inhalation of virus in larger droplets and subsequent culture-positive contagious infection. Air sampling was placed as close to the ferret as possible in a small chamber to increase the fraction of inhaled-to-delivered aerosol, but this approach is less exact than a nose-only exposure. Whole-body exposure for 1 h by unrestrained, unanaethetized ferrets was chosen to mimic the conditions of transmission experienced by humans in their daily activities. The advantages of this design include natural respiration patterns, expected dilution of airborne virus by deposition on the inanimate environment, and longer exposures to low concentrations of virus in the aerosol. The disadvantages include inability to exclude exposure by the non-inhalation routes, loss of variable amounts of aerosolized virus on chamber surfaces, and the need for surface decontamination between exposures. These disadvantages are advantages of the nose-only exposure systems [7], [8], [31], but the reduced minute ventilation of the ferret sleeping in the conical restraint may alter the amount or mucosal distribution of inhaled virus.

Detection of influenza virus in exhaled aerosols or nebulized aerosols presents multiple challenges. Humans exhale droplets of widely varying size and quantity [11]. Influenza RNA was detected in aerosols exhaled from 3 of 5 individuals infected with influenza A [5], and the exhaled virus was quantified as 20 RNA particles per minute in one subject. In a study capturing 3 cough specimens from ambulatory subjects with acute influenza A, 81% had viral RNA detected by M segment RT-qPCR in the cough droplets but only 2 of 21 had culturable virus [32]. We observed the same discrepancy between viable virus and viral RNA collection, with several possible explanations. Airborne virus has a very low death rate constant if the humidity is low [33] and this constant increases only modestly with increasing RH [34]. Exposures in this study were conducted at RH between 30–50%, and significant inactivation by humidity was not likely. Viral particles secreted into tissue culture media consists primarily of non-infectious particles with only 1% as infectious particles [35]–[37] and wide variation depending on cell source and culture conditions. Thus high levels of non-infectious viral particles nebulized from the stock solutions grown in tissue culture, and additional viral particle disruption due to high velocity passage through the Collison nebulizer jets, may dominate the collection at low levels of viral aerosol.

The accurate measurement of airborne virus depends on a number of experimental variables and the methods used to capture airborne virus. The PTFE filter is more efficient than the AGI impinger in capturing viral RNA but significantly less efficient in capturing infectious particles [38]. For brief intervals of collection we found the PTFE filter efficiently captured viable virus but after 20 min viability appeared to decrease but we demonstrated this only at high aerosolized virus levels. Indirect estimate of viable virus content using the ratio of infectious particle to viral RNA ratio was derived from the filter collections at high viral loads. Although this ratio is subject to variability, use of impinger collections to estimate the ratio was not valid due to the large loss of detectable viral RNA in impinger fluid.

For rapid high-throughput assessment of airborne influenza virus in environmental samples, culture may lack sufficient sensitivity. In this study the T5000™ Biosensor system demonstrated markedly higher sensitivity than standard RT-qPCR in samples expected to contain low levels of virus. The high specificity and sensitivity of Biosensor system has been published for diverse respiratory pathogens [22]–[24]. The breadth of coverage and resolution offered by this influenza panel was shown by testing 92 different influenza virus isolates, including 22 avian isolates (representing twenty different H/N types collected from nine different species of wild birds), 18 human influenza A isolates (eight H1N1, 10 H3N2), four swine isolates (including one novel type), one equine isolate, and six human influenza B isolates. Despite the diversity of this sample set, the broad-range primers generated amplicons from all isolates, while the base composition signatures from amplicons obtained with these primers distinguished the isolates [23]. In samples derived from clinical isolates at Northwestern University Hospital, the Biosensor system detected infection with approximately 94% sensitivity and 99% specificity. The detection of 6 genomic RNA segments by this assay may correlate sufficiently accurately with airborne virus levels and should be tested in environmental contamination studies.

The observation of persistent viral RNA in upper airway samples, seroconversion, yet negative cultures in ferrets may imply that aerosol transmission is not simply a dose-dependent event. Aerosol transmission may also depend on the behavior of the inhaled virus, due to strain-dependent virulence or the widely dispersed deposition of aerosolized virions. Humans infected with H3N2 strains are generally more symptomatic than those infected with seasonal H1N1 strains. Ferrets exposed to a seasonal H3N2 strain aerosol were infected by very low doses, shed infectious virus in upper airway secretions, and displayed typical symptoms [7], while ferrets in this study exposed to a range of aerosol doses of a seasonal H1N1 virus failed to produce airway virus or display symptoms. In our comparison of replication efficiency in human bronchial epithelial cells, this seasonal H1N1 strain produced 100-fold less infectious virus than the H1N1 2009 pandemic strain [39]. Moreover, in a comparison of ferret-to-ferret aerosol transmission of these two seasonal and pandemic H1N1 strains, the seasonal strain was not transmitted under experimental conditions permitting efficient transmission of the pandemic strain, despite the fact that the infected ‘donor’ ferrets exhaled higher levels of seasonal virus than pandemic virus (unpublished data). Furthermore, high levels of 2009 H1N1 vRNA>10^7^ Geq in nasal wash collections were accompanied by positive viral cultures >10^3^ FFU/collection. Thus aerosol transmission may be determined more by the innate strain-dependent replication efficiency in the susceptible host rather than the amount of virus exhaled by the contagious source.
